# The Effect of Perceived Social Support on Self-Esteem and Well-Being Among the General Population of Riyadh, Saudi Arabia

**DOI:** 10.7759/cureus.71454

**Published:** 2024-10-14

**Authors:** Bander A Haddad, Abdulrahman O Alomar, Lujain A Alleft, Nasser A Alkarney, Abeer T Bin Jumaiah, Shahad K Al-Ghanem, Osama S Alghamdi

**Affiliations:** 1 Psychiatry Department, College of Medicine, Al-imam Muhammed Ibn Saud University, Riyadh, SAU; 2 College of Medicine, Imam Mohammad Ibn Saud Islamic University, Riyadh, SAU; 3 College of Medicine, Royal College of Surgeons in Ireland, Dublin, IRL

**Keywords:** mental health, perceived social support, saudi arabia, self-esteem, well-being, who-5

## Abstract

Introduction

Perceived social support refers to the subjective assessment of the extent to which an individual feels supported by others. It is a crucial factor that affects a person's mental health, as well as poor self-esteem, which is a common psychiatric complaint correlated with low perceived social support. As the field expands, a better understanding of the correlation between these factors and mental health is important.

Aim

This study aimed to investigate the relationship between perceived social support, self-esteem, and well-being among the general population of Riyadh, Saudi Arabia.

Subjects and methods

This analytical cross-sectional study was conducted among the general population in Riyadh, Saudi Arabia. A self-administered questionnaire was distributed among the targeted population using an online survey. The survey comprised 39 questions, including a validated Arabic version of the Perceived Social Support (PSS) scale, a validated Arabic version of the Self-Esteem Scale, and the Well-Being Scale.

Results

Of the 405 surveyed participants, 277 (68.4%) were female, and 217 (53.6%) were between 18 and 25 years old. A total of 254 (62.7%) had high PSS. However, 328 (81.0%) had low self-esteem, and 236 (58.3%) had poor well-being. There was a significant association between PSS and both self-esteem and well-being. Females, those with higher monthly incomes, and those with good well-being were less likely to have low to moderate PSS levels. Additionally, being married and engaging in physical activity were significant predictors of low self-esteem.

Conclusion

Although most of the population demonstrated high PSS, the results for self-esteem and well-being were less than desired. PSS significantly influenced psychological well-being but not self-esteem. Further evidence suggests that male participants with lower monthly incomes may need more social support than others. More research is needed to determine the influence of PSS on self-esteem and well-being among the general population in this region.

## Introduction

Understanding social support is vital for grasping its impact on mental well-being. As this field of study expands, a better understanding of its effects has emerged. A study defined social support as the feeling of being important in the eyes of others, cared for and loved, respected as a human being, and having someone to help and listen to when needed [1]. The rationale for selecting perceived social support (PSS) instead of received social support stems from recognizing individual variations in social support levels. PSS refers to the subjective assessment of how much an individual feels supported. To avoid a narrow focus on quantitative metrics of social support, this study emphasizes perceived support, thereby prioritizing a clear understanding of the support individuals experience in their social networks.

Based on a literature review, low self-esteem and inadequate levels of well-being have been linked to social support. A 2016 study on medical professionals found a positive correlation between self-esteem levels and PSS [2]. Additionally, another study highlighted self-esteem as a major predictor of mental health [3]. Furthermore, demographic and social factors, such as being female or overweight, played a role in having low self-esteem [4]. A study investigating the correlation between PSS and self-esteem in 120 university graduates found that those with a higher perception of social support also had higher self-esteem, supporting previous findings [5]. However, one study concluded that there was no significant correlation between self-esteem and PSS, though both impacted participants' psychological well-being [6]. Regarding the association between psychological well-being and PSS, another study found that levels of depression, anxiety, and stress significantly affected an individual's well-being and were influenced by their PSS. The same study also concluded that PSS was a significant negative predictor of anxiety and depression [7]. Finally, in contrast to McClure et al., this study found no difference between male and female gender in PSS or self-esteem [8].

There is a clear lack of studies in Saudi Arabia and a limited understanding of the connection between well-being, self-esteem, and PSS. Therefore, this study aims to investigate the relationship between PSS, self-esteem, and well-being among the general population of Riyadh, Saudi Arabia, and to assess the association between demographic factors, self-esteem, and PSS.

## Materials and methods

Study design and participant enrollment

This analytical cross-sectional study was conducted from May 1, 2024, to September 27, 2024. It employed an online-based questionnaire to assess the association between PSS, self-esteem, and well-being. The study used self-selection sampling on adults from the general population of Riyadh, Saudi Arabia. A total of 405 participants were included, and the sample size was calculated using an online sample size calculator, ensuring a 95% confidence level with a 5% margin of error. Participants completed an online questionnaire via Google Forms, consisting of 39 questions, which was distributed through various online platforms (see appendix). The questionnaire, available in Arabic, incorporates validated Arabic scales, including the Multidimensional Scale of Perceived Social Support (Arabic-MSPSS) [9], the Arabic version of the Rosenberg Self-Esteem Scale (RSES), and the WHO Well-Being Scale. It also includes multiple demographic questions. Participants included in the study were adults aged 18 to 60 years residing in Riyadh, irrespective of ethnicity, who agreed to participate. Exclusions included individuals below 18 or over 60 years old, those residing outside of Riyadh, individuals who did not consent to participate, and those without online access.

Ethical consideration

The study was conducted in accordance with the ethical principles of the Declaration of Helsinki. Informed consent was obtained from all participants before their inclusion in the study. Data collection commenced after receiving ethical approval from the Local Committee for the Ethics of Research on Living Creatures at Imam Mohammad Ibn Saud Islamic University (IMSIU) (HAPO-01-R-011), with a consent code of 671/2024. The confidentiality of the anonymously collected data was maintained at all times, and the data were stored securely, accessible only to the researcher.

Measure

The Arabic-MPSS is a 12-item measure of PSS. Each item is rated on a 7-point Likert scale, ranging from "very strongly disagree" (coded as 1) to "very strongly agree" (coded as 7). A higher score indicates higher PSS [9]. The Arabic version of the MPSS was initially adapted to measure support from family, peers, and school personnel [10], and was later readapted to evaluate support from family, friends, and significant others. The mean scale score was stratified into three levels: scores ranging from 1 to 2.9 indicate low support; scores from 3 to 5 indicate moderate support; and scores from 5.1 to 7 indicate high support [11].

The RSES is composed of 10 items that assess self-perception by evaluating both positive and negative feelings. Responses are based on a 4-point Likert scale, ranging from 1 (strongly disagree) to 4 (strongly agree) [12]. Using the Arabic version, the score is calculated by summing the scores of all items after reversing the scores of items 2, 5, 6, 8, and 9. The total score ranges from 0 to 30, with scores between 15 and 25 considered within the normal range, and scores below 15 suggesting low self-esteem [13].

The WHO-5 Well-Being Index is a 5-item questionnaire that measures an individual's feelings over the past few weeks. Each item is rated on a 6-point Likert scale, ranging from "not at all" (coded as 0) to "always" (coded as 5). The raw score is calculated by totaling the five responses, with scores ranging from 0 to 25, where 0 represents the worst possible quality of life and 25 represents the best. To obtain a percentage score (ranging from 0 to 100), the raw score is multiplied by 4. A percentage score of 0 represents the worst possible quality of life, while a score of 100 represents the best. A cutoff point of ≤50 indicates poor well-being [14,15].

Statistical analysis

Categorical variables were presented as numbers and percentages, while continuous variables were calculated and reported as mean and standard deviation. The association between the PSS scale, self-esteem, and well-being was analyzed using the chi-square test. Additionally, the chi-square test was used to determine the relationship between PSS, self-esteem, well-being, and participants' sociodemographic data. Based on significant results, multivariate logistic regression analyses, using the enter method, were subsequently performed to identify significant independent predictors of low to moderate PSS, low self-esteem, and poor well-being. Statistical significance was set at p < 0.05. All data analyses were conducted using IBM SPSS Statistics for Windows, Version 26 (Released 2019; IBM Corp., Armonk, New York).

## Results

This study enrolled 405 participants. As shown in Table 1, 217 (53.6%) were between 18 and 25 years old, with females being the majority, 277 (68.4%). Students comprised 175 (43.2%) of the participants. Regarding marital status, 281 (69.4%) were single. In terms of education, 241 (59.5%) held a university degree. For family monthly income, 101 (24.9%) reported earning more than 30,000 SAR monthly. Most respondents, 355 (87.7%), were living with their families. Only 9 (2.2%) of the respondents indicated that their parents had divorced when they were below five years old. Additionally, 183 (45.2%) participated in individual sports.

**Table 1:** Sociodemographic characteristics of participants (n = 405) SAR: Saudi Arabian Riyal.

Study Variables	N (%)
Age group	
18–25 years	217 (53.6%)
26–35 years	95 (23.5%)
36–50 years	77 (19.0%)
51–60 years	16 (04.0%)
Gender	
Male	128 (31.6%)
Female	277 (68.4%)
Occupation	
Student	175 (43.2%)
Employed	169 (41.7%)
Unemployed	49 (12.1%)
Retired	12 (03.0%)
Marital status	
Single	281 (69.4%)
Married	107 (26.4%)
Divorced	17 (04.2%)
Educational level	
Uneducated	02 (0.50%)
Elementary school	06 (01.5%)
High school	102 (25.2%)
University	241 (59.5%)
Post-graduate	54 (13.3%)
Monthly family income (SAR)	
<10,000	88 (21.7%)
10,000–15,000	77 (19.0%)
15,001–20,000	86 (21.2%)
20,001–30,000	53 (13.1%)
>30,000	101 (24.9%)
Living status	
Living with family	355 (87.7%)
Living alone	41 (10.1%)
Living with a close friend	04 (01.0%)
Living with roommate	05 (01.2%)
Did the parents get divorced?	
No	371 (91.6%)
Yes, under 5 years old	09 (02.2%)
Yes, between the ages of 6–12 years	08 (02.0%)
Yes, between the ages of 13 and 18 years	09 (02.2%)
Yes, after 18 years	08 (02.0%)
Do you practice sports and what kind?	
I don't exercise	140 (34.6%)
Yes, individual sports	183 (45.2%)
Yes, team sports	22 (05.4%)
Yes, individual and team sports	60 (14.8%)

The PSS, self-esteem, and well-being index results are described in Table 2. The mean scores of the PSS subscales were 5.02, 5.27, and 5.43, representing the family, friend, and significant other subscale scores, respectively. The overall mean PSS score was 5.24 (SD: 1.17), with low, moderate, and high PSS levels constituting 16 (4.0%), 135 (33.3%), and 254 (62.7%) of the participants, respectively. Regarding the RSE scale, the overall mean RSE score was 9.40 (SD: 5.42), with 328 (81.0%) having low self-esteem and 77 (19.0%) having normal self-esteem. Finally, the total mean score for the well-being index was 44.3 (SD: 25.9), with 236 (58.3%) classified as having poor well-being and 169 (41.7%) classified as having good well-being.

**Table 2:** Descriptive statistics of perceived social support scale, self-esteem, and well-being scale (n = 405) WHO-5: 5-item World Health Organization.

Variables	Value
Perceive Social Support (PSS) Scale, mean ± SD	
Family subscale score	5.02 ± 1.29
Friend subscale score	5.27 ± 1.39
Significant other subscale score	5.43 ± 1.19
Overall PSS score	5.24 ± 1.17
Level of social support, N (%)	
Low (score 1–2.9)	16 (04.0%)
Moderate (score 3 to 5)	135 (33.3%)
High (5.1 to 7)	254 (62.7%)
Rosenberg Self-Esteem (RSE) Scale, mean ± SD	
RSE total score	9.40 ± 5.42
Level of self-esteem, N (%)	
Low (score <15)	328 (81.0%)
Normal (≥15)	77 (19.0%)
WHO-5 Well-Being Index, mean ± SD	
Well-being score	44.3 ± 25.9
Level of well-being, N (%)	
Poor (score ≤50)	236 (58.3%)
Good (score >50)	169 (41.7%)

**Figure 1:**
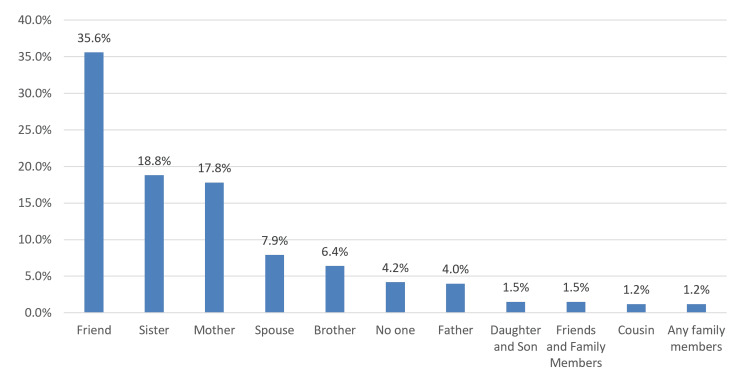
Special person who was perceived as a social support

Figure 1 illustrates that the most commonly perceived source of social support was a friend, reported by 144 participants (35.6%), followed by a sister (76 participants, 18.8%) and a mother (72 participants, 17.8%).

**Figure 2:**
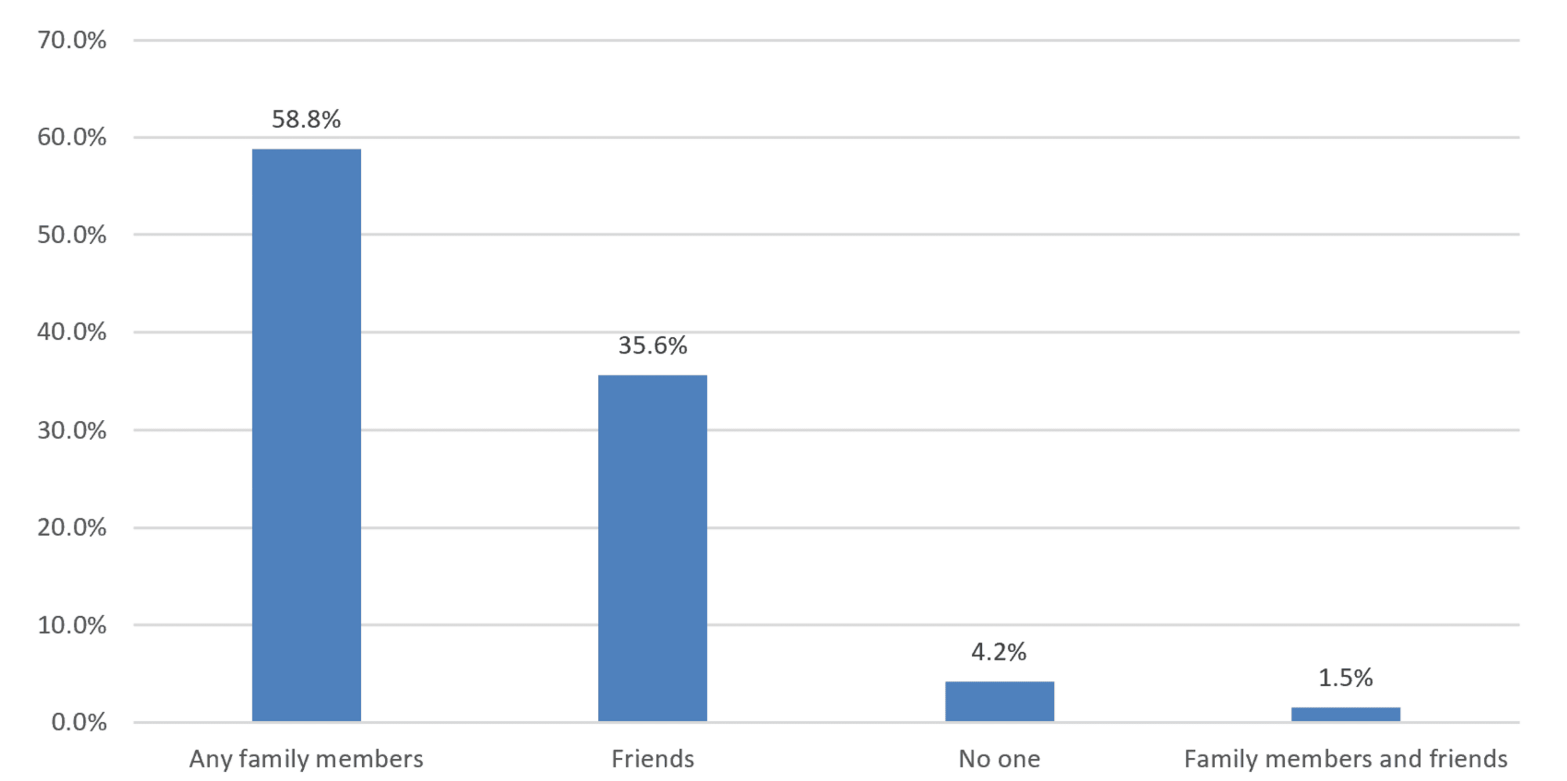
Groups who were perceived as a social support

Figure 2 illustrates that the most commonly perceived group for social support was family members, reported by 238 participants (58.8%), followed by friends (144 participants, 35.6%).

Measuring the association between PSS, self-esteem, and well-being revealed that higher levels of PSS were significantly associated with lower levels of self-esteem (p = 0.007) and better well-being (p = 0.001) (Table 3).

**Table 3:** Association between perceived social support in terms of self-esteem and well-being (n = 405) *P-value has been calculated using the chi-square test. **Significant at p<0.05 level.

Factor	Level of perceived social support		P-value*
	Low to moderate, n (%) (N = 151)	High, n (%) (N=254)	
Level of self-esteem			
Low	112 (74.2%)	216 (85.0%)	0.007**
Normal	39 (25.8%)	38 (15.0%)	
Level of well-being			
Poor	104 (68.9%)	132 (52.0%)	0.001**
Good	47 (31.1%)	122 (48.0%)	

When examining the relationship between PSS and the socio-demographic characteristics of participants (Table 4), we found that participants with higher PSS levels were more likely to be female (p = 0.040), married (p = 0.011), and have a better monthly income (p = 0.005). No significant relationships were observed between PSS levels and age group, occupation, educational level, living status, marital status of parents, or physical activity (p > 0.05).

**Table 4:** Relationship between perceived social support and participants' sociodemographic characteristics (n = 405) *P-value has been calculated using the chi-square test. **Significant at p<0.05 level.

Factor	Level of perceived social support		P-value*
	Low to Moderate n (%) (N = 151)	High n (%) (N=254)	
Age group			
≤25 years	83 (55.0%)	134 (52.8%)	0.666
>25 years	68 (45.0%)	120 (47.2%)	
Gender			
Male	57 (37.7%)	71 (28.0%)	0.040**
Female	94 (62.3%)	183 (72.0%)	
Occupation			
Unemployed	25 (16.6%)	36 (14.2%)	0.810
Student	64 (42.4%)	111 (43.7%)	
Employed	62 (41.1%)	107 (42.1%)	
Marital status			
Unmarried	122 (80.8%)	176 (69.3%)	0.011**
Married	29 (19.2%)	78 (30.7%)	
Educational level			
High school or below	43 (28.5%)	67 (26.4%)	0.646
University or higher	108 (71.5%)	187 (73.6%)	
Family monthly income (SAR)			
≤15,000	75 (49.7%)	90 (35.4%)	0.005**
>15,000	76 (50.3%)	164 (64.6%)	
Living status			
Living alone or non-family	16 (10.6%)	34 (13.4%)	0.409
Living with family	135 (89.4%)	220 (86.6%)	
Did the parents get divorced?			
No	137 (90.7%)	234 (92.1%)	0.624
Yes	14 (09.3%)	20 (07.9%)	
Physical activity			
No	56 (37.1%)	84 (33.1%)	0.411
Yes	95 (62.9%)	170 (66.9%)	

When measuring the relationship between self-esteem and the socio-demographic characteristics of participants (Table 5), it was revealed that lower levels of self-esteem were significantly associated with increasing age (p < 0.001), being employed (p = 0.011), being married (p < 0.001), and being physically active (p = 0.049). No significant relationships were observed between self-esteem levels and gender, education, monthly income, living status, or parents' marital status (p > 0.05).

**Table 5:** Relationship between self-esteem and participants' sociodemographic characteristics (n=405) *P-value has been calculated using the chi-square test. **Significant at p<0.05 level.

Factor	Level of self-esteem		P-value*
	Low, n (%) (N = 328)	Normal, n (%) (N = 77)	
Age group			
≤25 years	161 (49.1%)	56 (72.7%)	<0.001**
>25 years	167 (50.9%)	21 (27.3%)	
Gender			
Male	106 (32.3%)	22 (28.6%)	0.525
Female	222 (67.7%)	55 (71.4%)	
Occupation			
Unemployed	52 (15.9%)	09 (11.7%)	0.011**
Student	130 (39.6%)	45 (58.4%)	
Employed	146 (44.5%)	23 (29.9%)	
Marital status			
Unmarried	227 (69.2%)	71 (92.2%)	<0.001**
Married	101 (30.8%)	06 (07.8%)	
Educational level			
High school or below	90 (27.4%)	20 (26.0%)	0.795
University or higher	238 (72.6%)	57 (74.0%)	
Family monthly income (SAR)			
≤15,000	130 (39.6%)	35 (45.5%)	0.350
>15,000	198 (60.4%)	42 (54.5%)	
Living status			
Living alone or non-family	42 (12.8%)	08 (10.4%)	0.562
Living with family	286 (87.2%)	69 (89.6%)	
Did the parents get divorced?			
No	304 (92.7%)	67 (87.0%)	0.106
Yes	24 (07.3%)	10 (13.0%)	
Physical activity			
No	106 (32.3%)	34 (44.2%)	0.049**
Yes	222 (67.7%)	43 (55.8%)	

Exploring the relationship between the level of well-being and the socio-demographic characteristics of participants (Table 6), we observed that poor well-being was more prevalent among unmarried participants (p = 0.033). No significant relationships were found between well-being levels and age, gender, occupation, education, monthly income, living status, parents' marital status, or physical activity (p > 0.05).

**Table 6:** Relationship between well-being and participants' sociodemographic characteristics (n=405) *P-value has been calculated using the chi-square test. **Significant at p<0.05 level.

Factor	Level of well-being		P-value*
	Poor, n (%) (N = 236)	Good, n (%) (N = 169)	
Age group			
≤25 years	135 (57.2%)	82 (48.5%)	0.084
>25 years	101 (42.8%)	87 (51.5%)	
Gender			
Male	66 (28.0%)	62 (36.7%)	0.063
Female	170 (72.0%)	107 (63.3%)	
Occupation			
Unemployed	33 (14.0%)	28 (16.6%)	0.185
Student	111 (47.0%)	64 (37.9%)	
Employed	92 (39.0%)	77 (45.6%)	
Marital status			
Unmarried	183 (77.5%)	115 (68.0%)	0.033**
Married	53 (22.5%)	54 (32.0%)	
Educational level			
High school or below	65 (27.5%)	45 (26.6%)	0.838
University or higher	171 (72.5%)	124 (73.4%)	
Family monthly income (SAR)			
≤15,000	99 (41.9%)	66 (39.1%)	0.559
>15,000	137 (58.1%)	103 (60.9%)	
Living status			
Living alone or non-family	27 (11.4%)	23 (13.6%)	0.513
Living with family	209 (88.6%)	146 (86.4%)	
Did the parents get divorced?			
No	216 (91.5%)	155 (91.7%)	0.946
Yes	20 (08.5%)	14 (08.3%)	
Physical activity			
No	87 (36.9%)	53 (31.4%)	0.251
Yes	149 (63.1%)	116 (68.6%)	

Multivariate regression analyses, presented in Table 7, were performed to determine the significant independent predictors of low to moderate PSS, low self-esteem, and poor well-being. According to the results in model 1, compared to males, females had a 41% lower chance of having low to moderate PSS (adjusted odds ratio (AOR) = 0.594; 95% CI = 0.377-0.936; p = 0.025). Participants with a higher monthly income had a 45% lower chance of having low to moderate PSS compared to those with lower income levels (AOR = 0.551; 95% CI = 0.361-0.840; p = 0.006). Additionally, participants with good well-being were 48% less likely to have low to moderate PSS compared to those with poor well-being (AOR = 0.514; 95% CI = 0.330-0.811; p = 0.004). In model 2, married participants were 4.41 times more likely to have low self-esteem (AOR = 4.414; 95% CI = 1.621-12.020; p = 0.004). Physically active respondents were 1.88 times more likely to have low self-esteem levels (AOR = 1.880; 95% CI = 1.109-3.189; p = 0.019). In model 3, married participants had a lower risk of having poor well-being compared to unmarried participants (AOR = 0.617; 95% CI = 0.395-0.962; p = 0.033).

**Table 7:** Multivariate regression analysis to determine the significant independent predictors of low to moderate perceived social support, low self-esteem and poor well-being (n=405) **Significant at p<0.05 level. AOR: adjusted odds ratio, CI: confidence interval.

Variables	AOR	95% CI	P-value
Model 1: Perceived social support			
Gender			
Male	Ref		
Female	0.594	0.377–0.936	0.025**
Marital status			
Unmarried	Ref		
Married	0.675	0.404–1.129	0.135
Family monthly income (SAR)			
≤15,000	Ref		
>15,000	0.551	0.361–0.840	0.006**
Level of self-esteem			
Low	Ref		
Normal	1.512	0.883–2.589	0.132
Level of well-being			
Poor	Ref		
Good	0.517	0.330–0.811	0.004**
Model 2: Self-esteem			
Age group			
≤25 years	Ref		
>25 years	1.412	0.659–3.026	0.375
Occupation			
Unemployed	Ref		
Student	1.340	0.561–3.200	0.510
Employed	1.172	0.581–2.363	0.658
Marital status			
Unmarried	Ref		
Married	4.414	1.621–12.020	0.004**
Physical activity			
No	Ref		
Yes	1.880	1.109–3.189	0.019**
Model 3: Well-being			
Marital status			
Unmarried	Ref		
Married	0.617	0.395–0.962	0.033**

## Discussion

This study examined the relationship between PSS, self-esteem, and well-being among the general population of Riyadh, Saudi Arabia. Based on our extensive research, few studies in Saudi Arabia have investigated the impact of PSS on individuals' self-esteem and mental well-being. Therefore, the findings of this study will make a valuable contribution to the literature, given the importance of psychological well-being in our society.

Level of PSS

This study's findings revealed high PSS among the general population, with 254 (62.7%) participants, while 151 (37.3%) had low to moderate PSS (mean score: 5.24 ± 1.17). Among PSS subscales, significant others showed the highest mean score (5.43), and family subscales had the lowest mean score (5.02). This is not consistent with the study of Anque and Ceballo. Using the MPSS questionnaire, the level of PSS, including its subscales (family, friends, and significant others), among senior high school students was deemed moderate, which was lower than our reports [6]. This corroborates one other study, where more than half of students (54%) reported receiving moderate social support [16]. Individuals receiving appropriate social support can handle difficulties and challenges better. Hence, social interaction among individuals who lack social support is imperative.

Demographic factor of PSS

The chi-square test revealed that gender, marital status, and family monthly income significantly influenced PSS (p < 0.05). However, our multivariate regression estimates showed that only gender and monthly income remained significant. The model suggests that females and participants with higher incomes were associated with better social support than their male counterparts with lower monthly income. Our results are more favorable compared to the study by Tam et al., which reported no significant differences in PSS based on gender (p > 0.05) [8]. Similarly, Asif found that PSS did not differ by gender in family settings, but it varied among friends [17].

Level of self-esteem

Despite the majority receiving high social support, more than 80% of our population was regarded as having low self-esteem (mean score 44.3 out of 100 points). While our subjects exhibited low self-esteem, senior students in the Philippines showed better results, with the majority demonstrating high self-esteem levels [6]. A study conducted among 500 Egyptian students presented a similar scenario, with approximately two-thirds having moderate self-esteem and only 4.6% having low self-esteem. Identifying the cause of low self-esteem in our population is necessary to address this gap. Further investigation may help find the underlying causes and initiate necessary interventions within the population in our region.

Demographic factor of self-esteem

Based on our univariate analysis, increasing age, marriage, employment, and physical activity were associated with low self-esteem. However, in our multivariate regression model, being married (AOR = 4.41) and engaging in physical activity (AOR = 1.88) remained significant, identified as the independent predictors of low self-esteem. This is not consistent with the findings of McClure et al., where demographic factors such as Hispanic race, higher body mass index, and daily TV time were significant predictors of low self-esteem. In contrast, black teens with better school performance or engagement in team sports had a lower chance of reporting low self-esteem [4]. These variations may be attributed to differences in regional settings, population diversity, and age groups.

Level of well-being

Based on the WHO-5 criteria [14], nearly sixty percent of our population was considered to have poor well-being (mean score 44.3 out of 100 points). This is lower than the findings of Anque & Ceballo, which suggest that high school students have higher psychological well-being [6]. In contrast, a study in Pakistan documented that PSS in students predicted 6% of depression and 2% of anxiety, with the majority of students experiencing mild to severe levels of depression, anxiety, and stress [7]. Poor well-being can lead to multiple comorbid conditions, emphasizing the need for interventions to improve well-being. Coping strategies such as social interactions, enhanced self-esteem, and engagement in physical activity could improve the psychological well-being of the population.

Demographic factor of well-being

Data from our study suggest that marital status was the only factor influencing well-being. Specifically, unmarried individuals were at an increased risk of poor well-being, while married individuals were associated with better mental well-being. This contrasts with the findings of Auttama et al., which suggest that associated diseases and family relationships greatly influence mental health issues. In their study, female gender, normal weight, and a better relationship with family were more closely associated with better psychological self-care [3].

Relationship between PSS, self-esteem, and well-being

Our results highlight the influence of well-being on PSS. Based on our adjusted regression model, respondents with low to moderate PSS had a decreased chance of having good well-being. However, self-esteem did not significantly affect PSS after adjustments for potential confounders. This is comparable to a study conducted in Chile, which reported that students with a better balance of affection were associated with a higher perception of social support. Furthermore, the study emphasized that improving the perception of social support could enhance students' eudaimonic well-being (pursuing happiness by finding meaning and purpose) [18]. In contrast, a study among Danish students found that social support from peers was inversely associated with perceived stress but positively correlated with life satisfaction, influenced by the interaction of self-esteem and self-efficacy [19].

Study limitations

This study has several limitations that affect the generalizability of the findings. First, the distribution of males versus females was not balanced, limiting the ability to generalize the results of gender comparisons related to the outcome variables. Second, the survey was conducted using an online platform, which may have discouraged respondents from providing accurate and honest responses, potentially introducing recall bias. Measures such as setting the upper age limit to 60 were implemented to reduce recall bias due to benign senescent forgetfulness, dementia, and other conditions. Third, various biases, including selection bias, sampling bias, and non-response bias, could affect the generalizability of our findings. These biases highlight the importance of careful study design, appropriate data collection methods, and the use of statistical techniques to adjust for potential confounders. Finally, being a cross-sectional study, it is prone to bias and does not establish cause-and-effect relationships.

## Conclusions

This study provides evidence that individuals with low to moderate social support are associated with poor well-being, while self-esteem remained consistently low across the population. Female participants with higher monthly incomes reported better social support than others, and married participants exhibited better mental well-being but lower self-esteem. The findings of this study can assist policymakers in developing interventions aimed at improving the population's well-being. Promoting greater social interaction could enhance psychological well-being within the community.

## Appendices

Study questionnaire

The study questionnaire is given in Table 8.

**Table 8:** Sociodemographic questionnaire

Age group
18–25 years
26–35 years
36–50 years
51–60 years
Gender
Male
Female
Occupation
Student
Employed
Unemployed
Retired
Marital status
Single
Married
Divorced
Educational level
Uneducated
Elementary school
High school
University
Post-graduate
Monthly family income (SAR)
<10,000
10,000–15,000
15,001–20,000
20,001–30,000
>30,000
Living status
Living with family
Living alone
Living with a close friend
Living with roommate
Did the parents get divorced?
No
Yes, under 5 years old
Yes, between the ages of 6–12 years
Yes, between the ages of 13 and 18 years
Yes, after 18 years
Do you practice sports and what kind?
I don't exercise
Yes, individual sports
Yes, team sports
Yes, individual and team sports

Multidimensional scale of perceived social support

The multidimensional scale of perceived social support is given in Table 9.

**Table 9:** Multidimensional scale of perceived social support

Instructions: We are interested in how you feel about the following statements. Read each statement carefully. Indicate how you feel about each statement.							
Question	Very Strongly Disagree	Strongly Disagree	Mildly Disagree	Neutral	Mildly Agree	Strongly Agree	Very Strongly Agree
1. There is a special person who is around when I am in need.							
2. There is a special person with whom I can share joys and sorrows.							
3. My family really tries to help me.							
4. I get the emotional help & support I need from my family.							
5. I have a special person who is a real source of comfort to me.							
6. My friends really try to help me.							
7. I can count on my friends when things go wrong.							
8. I can talk about my problems with my family.							
9. I have friends with whom I can share my joys and sorrows.							
10. There is a special person in my life who cares about my feelings.							
11. My family is willing to help me make decisions.							
12. I can talk about my problems with my friends							
Who is the special person you mentioned in the scale above:							

Rosenberg Self-Esteem Scale (RSE)

The Rosenberg Self-Esteem Scale (RSE) questionnaire is shown in Table 10.

**Table 10:** Rosenberg Self-Esteem Scale (RSE)

Instructions: Please check the appropriate answer box for each item, depending on whether you Strongly agree, agree, disagree, or strongly disagree with it.				
Question	Strongly agree	Agree	Disagree	Strongly disagree
1. On the whole, I am satisfied with myself.				
2. At times I think I am no good at all.				
3. I feel that I have a number of good qualities.				
4. I am able to do things as well as most other people.				
5. I feel 1do not have much to be proud of.				
6. I certainly feel useless at times.				
7. I feel that I'm a person of worth.				
8. I wish I could have more respect for myself.				
9. All in all, I am inclined to think that I am a failure.				
10. I take a positive attitude toward myself.				

WHO-5 Well-Being Index

The WHO-5 Well-Being Index questionnaire is shown in Table 11.

**Table 11:** WHO-5 Well-Being Index

Please respond to each item by marking one box per row, regarding how you felt in the last two weeks.		All of the time	Most of the time	More than half the time	Less than half the time	Some of the time	At no time
WHO 1	I have felt cheerful in good spirits.						
WHO 2	I have felt calm and relaxed.						
WHO 3	I have felt active and vigorous.						
WHO 4	I woke up feeling fresh and rested.						
WHO 5	My daily life has been filled with things that interest me.						
